# Regorafenib induces Bim-mediated intrinsic apoptosis by blocking AKT-mediated FOXO3a nuclear export

**DOI:** 10.1038/s41420-023-01338-9

**Published:** 2023-01-31

**Authors:** Beini Sun, Hongce Chen, Xiaoping Wang, Tongsheng Chen

**Affiliations:** 1 MOE Key Laboratory of Laser Life Science & Guangdong Provincial Key Laboratory of Laser Life Science, College of Biophotonics, South China Normal University, Guangzhou, 510631 China; 2 Department of Pain Management, The First Affiliated Hospital of Jinan University, Guangzhou, 510632 China; 3 SCNU Qingyuan Institute of Science and Technology Innovation Co., Ltd., South China Normal University, Qingyuan, 511500 China

**Keywords:** Apoptosis, Targeted therapies

## Abstract

Regorafenib (REGO) is a synthetic oral multi-kinase inhibitor with potent antitumor activity. In this study, we investigate the molecular mechanisms by which REGO induces apoptosis. REGO induced cytotoxicity, inhibited the proliferation and migration ability of cells, and induced nuclear condensation, and reactive oxygen species (ROS)-dependent apoptosis in cancer cells. REGO downregulated PI3K and p-AKT level, and prevented FOXO3a nuclear export. Most importantly, AKT agonist (SC79) not only inhibited REGO-induced FOXO3a nuclear localization and apoptosis but also restored the proliferation and migration ability of cancer cells, further demonstrating that REGO prevented FOXO3a nuclear export by deactivating PI3K/AKT. REGO treatment promotes Bim expression via the FOXO3a nuclear localization pathway following PI3K/AKT inactivation. REGO induced Bim upregulation and translocation into mitochondria as well as Bim-mediated Bax translocation into mitochondria. Fluorescence resonance energy transfer (FRET) analysis showed that REGO enhanced the binding of Bim to Bak/Bax. Knockdown of Bim, Bak and Bax respectively almost completely inhibited REGO-induced apoptosis, demonstrating the key role of Bim by directly activating Bax/Bak. Knockdown of Bax but not Bak inhibited REGO-induced Drp1 oligomerization in mitochondria. In conclusion, our data demonstrate that REGO promotes apoptosis via the PI3K/AKT/FOXO3a/Bim-mediated intrinsic pathway.

## Introduction

Regorafenib (REGO) is a synthetic potent oral multi-kinase inhibitor against a series of proteins involved in tumorigenesis (KIT, RET, and RAF), angiogenesis (VEGFR1-3 and TIE2), and maintenance of the tumor microenvironment (PDGFR and FGFR) protein kinase inhibitory [1–3]. REGO also destroys tumor immunity and survival by inhibiting the colony-stimulating factor 1 receptor (CSF-1R), which is important for macrophage differentiation, and leads to a decrease of tumor-infiltrating macrophages [4]. Because of its broad-spectrum kinase inhibition, REGO has been clinically proved more effective antitumor activity than other specific angiogenesis inhibitors [5]. In clinical trials, REGO has been used to treat metastatic colorectal cancer (CRC), gastrointestinal stromal tumors, liver cancer, and other malignant tumors (such as lung, esophageal cancer) [6–8]. Although REGO is similar in structure to sorafenib, only one fluorine atom is added to the central benzene ring, which makes it have different biochemical and pharmacological properties to sorafenib [3].

Phosphatidylinositol 3-kinase (PI3K) plays a key role in tumorigenesis and progression, and it is considered one of the key targets for cancer therapy [9]. And protein kinase B (AKT) has been widely studied as a major player in PI3K signal transduction. PI3K/AKT pathway plays a key role in many cell functions, including proliferation, migration, invasion, adhesion, metabolism and survival [10, 11]. However, PI3K/AKT signaling pathway is deregulated in almost all cancers, commonly found in breast cancer, ovarian cancer, head and neck cancer, lung cancer, colorectal cancer, and bladder cancers [12, 13], and they are also considered to be central sensors of carcinogenic signals in the progress of tumors [14]. Forkhead box O (FOXO) has been shown to be a core downstream player in the PI3K/AKT signaling pathway [15]. AKT leaves FOXO in cytoplasm, and loss of survival factors leads to FOXO nuclear translocation and thus activation of target genes. Bim is one of Foxo3a’s most important target genes in the nucleus [16, 17]. The PI3K/AKT/FOXO3a signaling hub has also been shown to play an important role in many other chemotherapeuticses, such as 18β-glycyrrhetinic acid, quercetin, purpuriniso, the Novel Benzothiazole Derivative PB11, flavone and paclitaxel [18–23].

Pro-apoptotic BH3-only proteins of Bcl-2 family proteins (e.g., activators Bid, Bim and Puma and sensitizers Bad, Bik, BMF and NOXA) play a very critical role in cancer cell apoptosis [24]. Activators can bind directly to Bax/Bak and activate them, causing them to oligomerization on mitochondria, which leads to mitochondrial outer membrane permeabilization (MOMP), release of apoptotic factors and activation of caspases 3/9, thus causing apoptosis [25, 26]. Bim is highly pro-apoptotic by initiating intrinsic apoptotic pathways under physiological and pathophysiological conditions. Bim expression can be transcriptionally regulated by a variety of transcription factors, post transcriptionally regulated is mainly through alternative splicing and binding to microRNA, and translated regulation is mainly through open reading frameworks as well as phosphorylation and degradation, and spatially located and isolated [27, 28]. Bim expression is related to cancer cell inhibition, tumor promotion, metastasis and drug resistance, so it has attracted attention on chemotherapy [29, 30]. A Bim gene mutation in some East Asian populations plays an important role in the drug resistance of EGFR-mutated lung cancer [31]. A variety of chemotherapeutics drugs, such as doxorubicin, paclitaxel, and tinib, upregulated Bim expression in breast, colon, lung, and leukemia [29, 32]. It was shown that Bim is a key regulator of apoptosis following oncogene inactivation in multiple transgenic mouse models of oncogene (MYC, BCR-ABL, RAS)-induced acute lymphoblastic leukemia [33]. However, whether Bim is still a key factor in mediating apoptosis among multi-kinase inhibitors has not been reported.

In this study, we found that REGO inhibited the proliferation of various cancer cell lines. Then we studied the molecular mechanism by which REGO induces apoptosis in MCF-7 cells. It is proved for the first time that REGO induces apoptosis through the PI3K/AKT/FOXO3a signal pathway in MCF-7 cells. REGO promotes nuclear translocation of FOXO3a by inhibiting PI3K and AKT phosphorylation to increase Bim expression, which in turn activates Bax and Bak. Bax, not Bak, recruited dynamin-related protein 1 (Drp1) to transport mitochondria, leading to mitochondrial fragmentation and eventual apoptosis. These findings provide the basis for new ideas and mechanisms for the use of REGO in cancer therapy.

## Results

### REGO inhibits cell proliferation and colony formation and induces apoptosis in human cancer cells

**Fig. 1:**
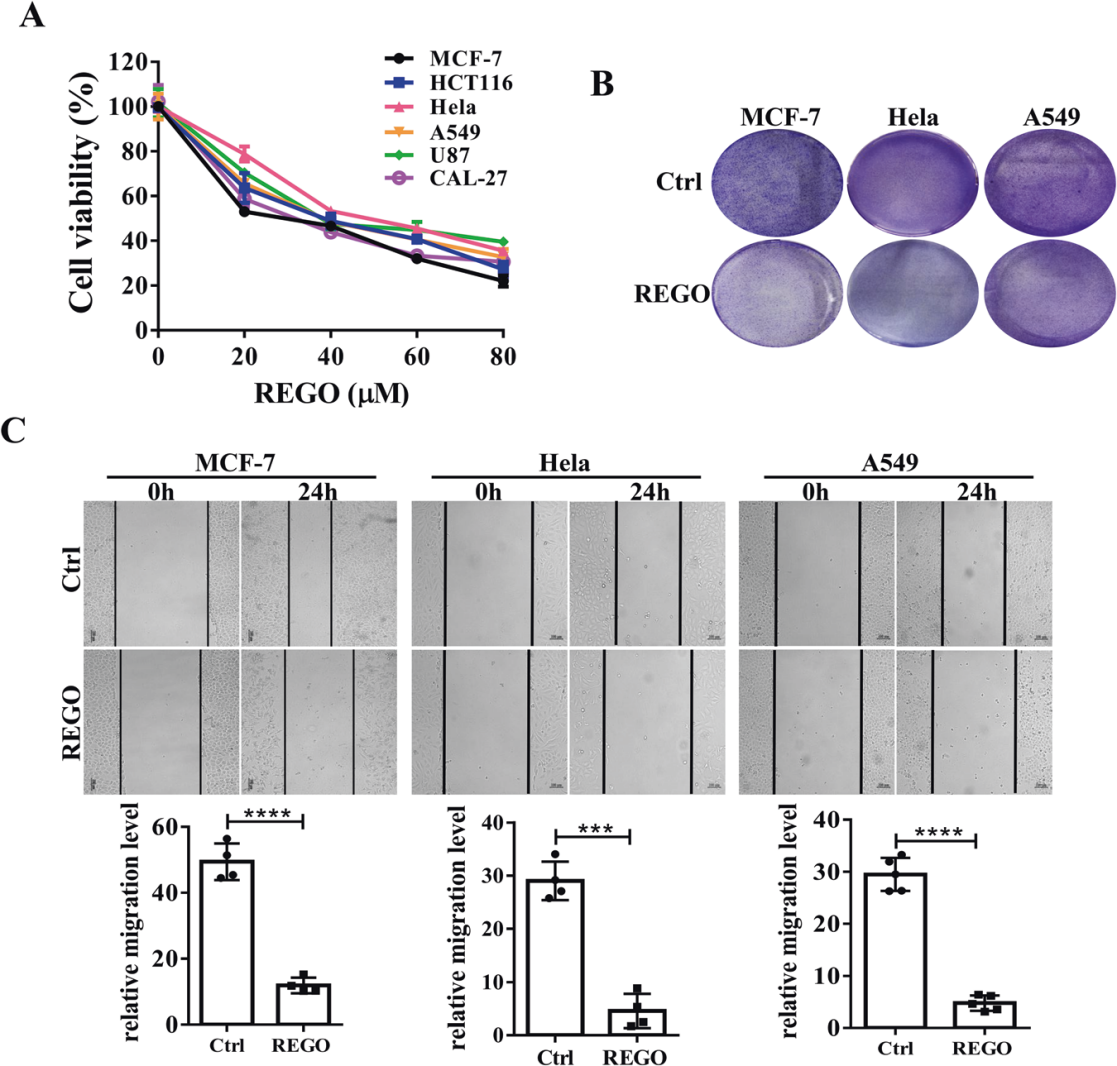
REGO inhibits cell proliferation and migration in human cancer cells. **A** REGO induced dose-dependent cytotoxicity in six tumor cell lines measured by CCK-8 assay. **B** The ability of REGO-inhibited proliferation of MCF-7, Hela and A549 cells assessed by cell colony formation assay. **C** The ability of REGO-induced MCF-7, Hela and A549 cells migratory capability evaluated by the wound healing assays. Representative images of MCF-7, Hela and A549 cells are shown as photomicrographs (×100). ***p < 0.001, ****p < 0.0001. All data are expressed with the mean ± SD of three independent experiments.

To evaluate the effects of REGO on the growth of human cancer cells, the cell viability of various human cancer cells, including MCF-7 breast cancer cells, HCT116 colorectal cancer cells, Hela cervical cancer cells, A549 non-small-cell lung cancer cells, U87 brain glioma cells, and CAL-27 human tongue squamous cell carcinoma cell line, were determined by CCK-8 assay. The IC50 of REGO in MCF-7, HCT116, Hela, A549, U87, and CAL-27 cell lines were 25.37 ± 0.21 µM, 33.33 ± 1.54 µM, 36.76 ± 0.96 µM, 34.25 ± 1.97 µM, 34.26 ± 1.23 µM and 26.24 ± 2.07 µM, respectively. We found that in these cancer cells, REGO decreased cell survival in a dose-dependent manner (Fig. 1A). We also tested the effect of REGO on the in vitro migration and antiproliferative effect of MCF-7, Hela and A549 cells by wound-healing assays and cell colony formation assays. As shown in Fig. 1B, C treating MCF-7, Hela and A549 cells with REGO significantly decreased the number of colonies and migration ability. These results suggest that REGO inhibits the ability of human cancers to proliferation and migration.

**Fig. 2:**
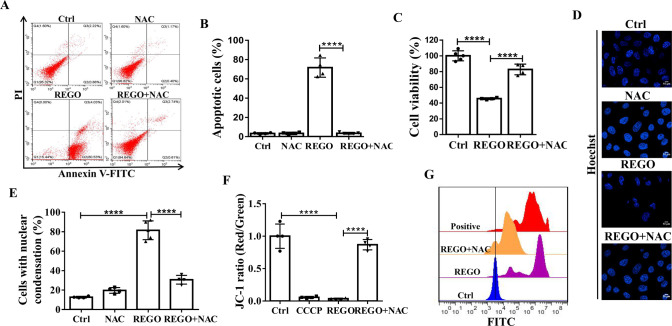
REGO induces ROS-dependent apoptosis in MCF-7 cells. **A**, **B** Flow cytometry analysis on REGO-induced apoptosis. **C** REGO induced ROS-dependent cytotoxicity measured by CCK-8 assay. Cells were pretreated with NAC (10 mM) for 2 h, followed by co-treatment with REGO at 20 μM for 24 h. **D** REGO induced ROS-dependent nuclear condensation detected by Hoechst 33258 staining (×600). **E** Statistical percentages of cells with nuclear condensation. *N* ≥ 100 cells. **F** REGO-induced loss of mitochondrial membrane potential by using JC-1 probe. **G** REGO significantly increased intracellular ROS. **p < 0.01, ***p < 0.001, ****p < 0.0001. All data are expressed with the mean ± SD of three independent experiments.

We next quantitatively analyzed Annexin-V/PI-stained cells by flow cytometry to verify whether REGO induces apoptosis. REGO treatment significantly increased apoptotic cells, which was markedly reduced by NAC (a reactive oxygen species (ROS) inhibitor) treatments (Fig. 2A, B). By CCK-8 assay and Hoechst staining, we found that NAC significantly inhibited REGO-induced nuclear condensation and cell death (Fig. 2C–E). JC-1 probe was used to examine whether REGO causes mitochondrial membrane potential loss. Fluorescence microscopic imaging showed that REGO induced a significant decrease in mitochondrial membrane potential, and NAC inhibited significantly the REGO-induced decrease in membrane potential (Fig. 2F). REGO also induced a significant increase in intracellular ROS, which was significantly inhibited by NAC pretreatment (Fig. 2G). In order to further observed the production of ROS, we detected mitochondrial ROS (mROS) by MitoSOX probe. Fluorescence microscopic imaging showed that REGO induced the production of mROS (Fig. S1A). By CCK-8 analysis, we found that MitoTEMPO (mROS inhibitor) significantly inhibited REGO-induced cell death (Fig. S1B). To test whether REGO-induced mROS accumulation by inhibition of superoxide dismutase 2 (SOD2). We detected the mRNA expression of SOD2 by QPCR. Compared to WT cells, REGO treatment significantly decreased SOD2 via FOXO3a (Fig. S1C and Table S1). These results suggest that REGO induces ROS-dependent apoptosis.

### REGO induces apoptosis by inhibiting the PI3K/AKT/Foxo3a signaling pathway

**Fig. 3:**
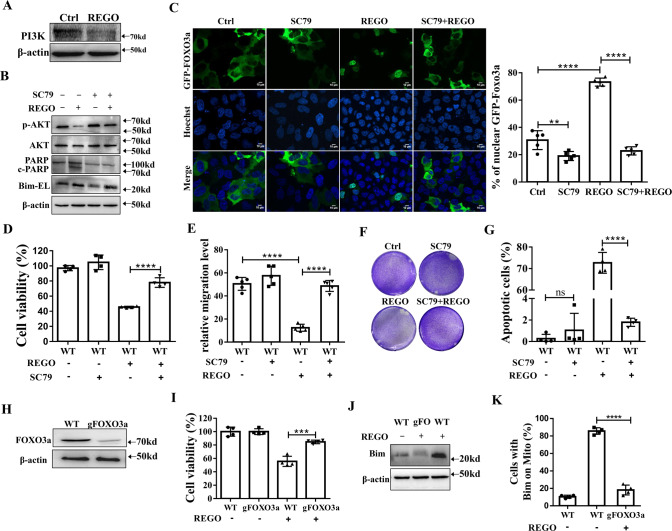
REGO induces apoptosis by inhibiting the PI3K/AKT/FOXO3a signaling pathway. **A** Western blotting analysis on the REGO-down-regulated PI3K. **B** Western blotting analysis on the inhibitory effect of SC79 on REGO. Cells were pretreated with SC79 (10 mM) for 2 h, followed by co-treatment with REGO at 20 μM for 24 h. **C** Representative fluorescence images of cells expressing GFP-FOXO3a in the presence or absence of REGO. Scale bar: 10 μm. Statical percentage of cells with GFP-FOXO3a nuclear localization. *n* ≥ 100 cells. **D** Inhibitory effect of SC79 on the cytotoxicity of REGO measured by CCK-8 assay. Cells were pretreated with SC79 (10 mM) for 2 h, followed by co-treatment with REGO at 20 μM for 24 h. **E** SC79 prevents the REGO-induced loss of cells migration assessed by wound healing assay. Representative images of MCF-7 cells are shown as micrographs (×100). **F** SC79 restores REGO-induced proliferation of MCF-7 cells was assessed by a cell colony formation assay. **G** Flow cytometry analysis on the inhibitor effects of SC79 on REGO-induced apoptosis. **H** Western blotting analysis confirmed CRISPR/Cas9-mediated knockout of FOXO3a. **I** FOXO3a was required for REGO-induced cytotoxicity measured by CCK-8 assay. **J** Western blotting analysis on FOXO3a was involved in REGO-triggered Bim expression. **K** Statistical percentage of cells mitochondrial YFP-Bim on. *n* ≥ 100 cells. **p < 0.01, ***p < 0.001, ****p < 0.0001, ns (no significant) *p* > 0.05. All data are expressed with the mean ± SD of three independent experiments.

The PI3K/AKT/FOXO3a pathway plays a critical role in apoptosis and cell proliferation [34]. To determine the roles of The PI3K/AKT/FOXO3a pathway in REGO-induced apoptosis in MCF-7 cells, we used western blotting analysis to determine the protein levels of factors involved in PI3K/AKT/FOXO3a signaling. REGO treatment significantly reduced the expression levels of PI3K and p-AKT (Fig. 3A, B). Next, we investigated the effect of AKT on REGO efficacy, and AKT agonist (SC79) was used to pre-treat cells. Western blotting assays showed that compared with the REGO group, SC79 pretreatment increased p-AKT expression, decreased Bim expression, and completely prevented cleaved-PARP (Fig. 3B). We therefore explored whether FOXO3a nuclear-cytoplasmic translocation was influenced by REGO. Cells were transfected with GFP-FOXO3a. As shown in Fig. 3C, REGO treatment significantly induced GFP-FOXO3a nuclear localization, whereas SC79 significantly inhibited REGO-induced GFP-FOXO3a nuclear localization. These results indicated that REGO induced FOXO3a nuclear localization which was inhibited by SC79. CCK-8 was used to evaluate the effect of SC79 treatment on cell viability. SC79 inhibited significantly REGO-induced cytotoxicity in MCF-7 cells (Fig. 3D). We also examined the effect of AKT on the migration and proliferation of the REGO-induced cells by wound-healing assays and cell colony formation assays. As shown in Figs. 3E, F and S2A SC79 restored significantly the migratory and proliferative abilities of REGO-treated MCF-7 cells. Flow cytometry Annexin-V/PI staining analysis further suggested that SC79 significantly inhibited REGO-induced apoptosis (Figs. 3G and S2B). We constructed the gFOXO3a-transfected (gFOXO3a) cell line by CRISPR/Cas 9 technology. Western blotting analysis confirmed the genetic deletion of FOXO3a (Fig. 3H). Then, we used CCK-8 to detect the viability of gFOXO3a cells treated with REGO, respectively. Compared with wild-type (WT) cells, gFOXO3a significantly inhibited REGO-induced cytotoxicity (Fig. 3I). To further demonstrate whether Bim activation was associated with REGO-induced FOXO3a nuclear localization, western blotting and QPCR analysis showed that the gFOXO3a group decreased Bim expression compared to the REGO group (Figs. 3J and S2C). We transfected YFP-Bax in WT and gFOXO3a cells, stained cells with Mitotracker Deep Red 633, and observed post-transfection YFP-Bim using an Apotome 2 microscope. In control cells, YFP-Bim uniformly distributed, while in the REGO-treated group, YFP-Bim was significantly accumulated in mitochondria, and in gFOXO3a cells, the mitochondrial distribution and aggregation of YFP-Bim were significantly decreased (Figs. 3K and S2D). Further, we detected endogenous FOXO3a by immunofluorescence. REGO-induced FOXO3a nuclear localization which was inhibited by SC79 (Fig. S2E). Eliminate the potential influence of gFOXO3a on the expression of other FOXO factors. We detected the expression of other FOXO factors by QPCR, and compared with WT cells, gFOXO3a cells showed no difference in the expression of other FOXO factors (Fig. S2F). In order to further explain that enhanced nuclear localization of FOXO3a leads to decreased cell viability and migration in MCF7 cells, we constructed ectopic expression of a PKB-insensitive FOXO3a triple alanine (A3) mutant. CCK-8 and wound healing assays were used to analyze the effect of SC79 treatment on cell viability and migration ability. FOXO3a A3 leads to the a decrease in cell viability and migration ability, and was not inhibited by SC79 (Fig. S2G, H). Taken together, these results demonstrate that REGO induces apoptosis by inhibiting the PI3K/AKT/FOXO3a signaling pathway.

### Bim, Bax and Bak mediate REGO-induced apoptosis

**Fig. 4:**
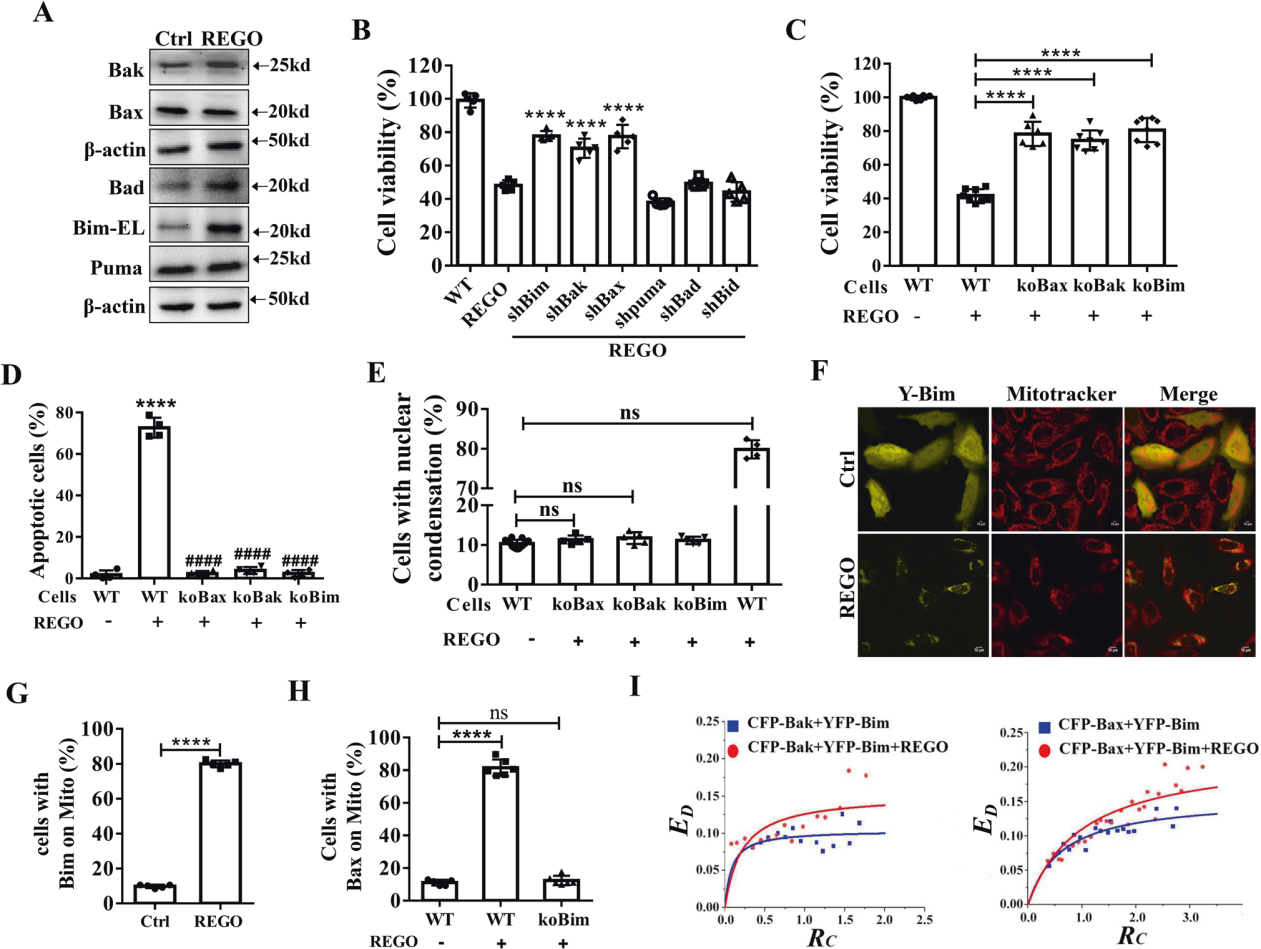
Bim mediates REGO-induced intrinsic apoptosis. **A** Western blotting analysis on REGO-regulated the expression of apoptosis-related proteins as indicated. **B** Roles of Bcl-2 family proteins in REGO-induced cytotoxicity by silencing the indicated proteins (compared with the REGO group). **C** Bax, Bak and Bim were required for REGO-induced cytotoxicity measured by CCK-8 assay. **D** Flow cytometry analysis on the key role of Bax, Bak and Bim in REGO-induced apoptosis (* compared with the control group, # compared with the REGO group). **E** Statistical percentage of cells with nuclear condensation. *n* ≥ 500 cells. **F**, **G** Representative fluorescence images of cells expressing YFP-Bim in the presence or absence of REGO (×600). Statistical percentage of cells mitochondrial YFP-Bim on. *n* ≥ 100 cells. **H** Bim-mediated REGO-induced Bax translocation into mitochondria. Statistical percentage of transfected cells with the mitochondrial distribution of Bax. **I** Quantitative FRET measurements in living cells coexpressing CFP-Bak and YFP-Bim or CFP-Bax and YFP-Bim and the corresponding *E*_*D*_–*R*_*C*_ plot. **F** Western blotting analysis on REGO-regulated the expression of Bcl-xl. *p < 0.05, ***p < 0.001, ****p < 0.0001. All data are expressed with the mean ± SD of three independent experiments.

**Fig. 5:**
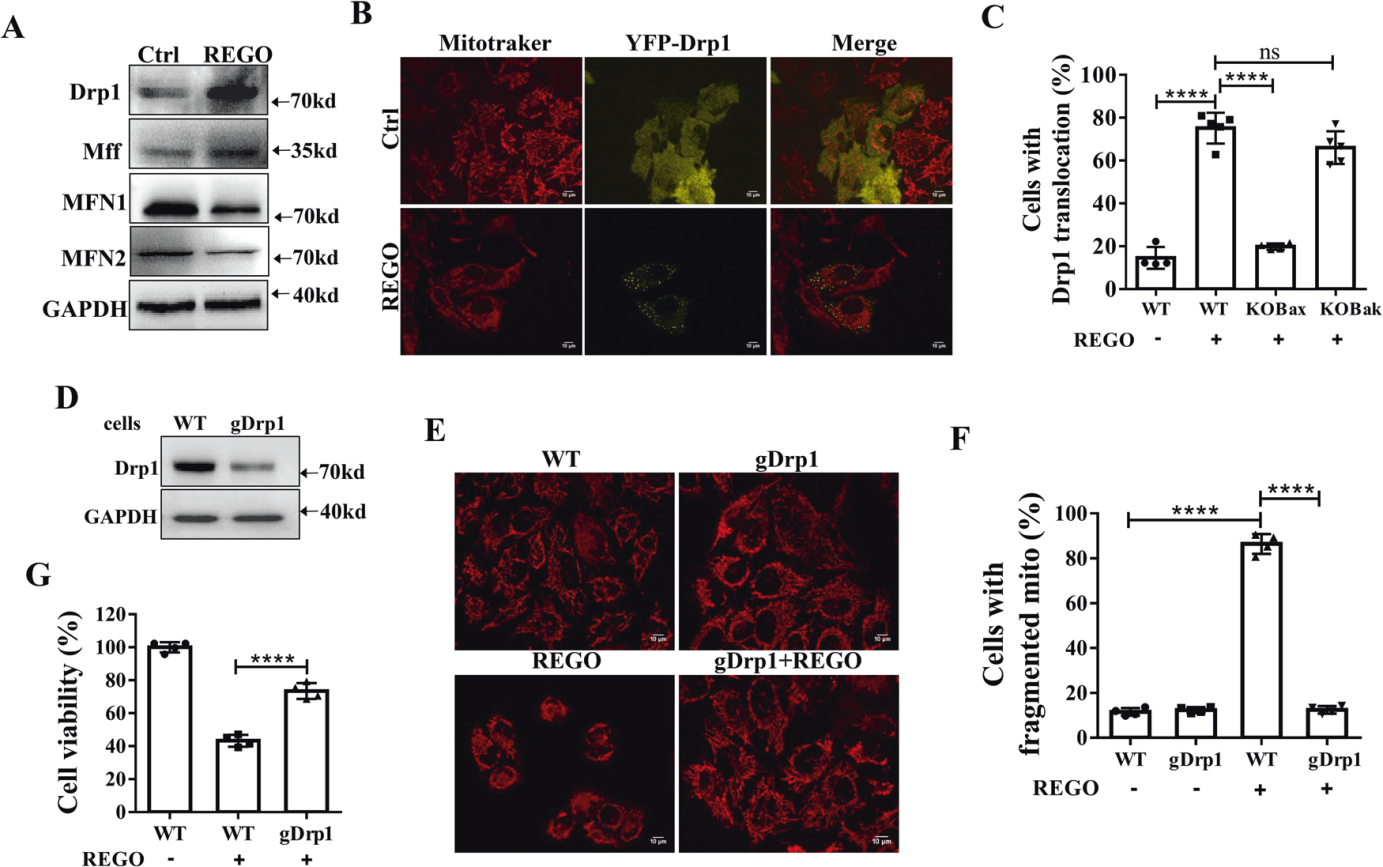
Bax, but not Bak, recruits Drp1 to mitochondria to mediate REGO-induced mitochondrial fission. **A** Western blotting analysis on REGO-regulated the expression of mitochondrial fusion and division-related proteins as indicated. **B** Representative fluorescence images of cells expressing YFP-Drp1 in the presence or absence of REGO (×600). **C** Statistical percentage of cells with mitochondrial Drp1. **D** Western blotting analysis confirmed CRISPR/Cas9-mediated knockout of Drp1. **E** Representative fluorescence images of intracellular mitochondrial morphology of WT and gDrp1 in the presence or absence of REGO (×600). **F** Statistical percentage of cells with fragmented mitochondria. **G** Drp1 was required for REGO-induced cytotoxicity measured by CCK-8 assay. *N* ≥ 50 cells. ****p < 0.0001, ns (no significant) *p* > 0.05.

To investigate the role of Bcl-2 family proteins in REGO-induced apoptosis, we assessed the effect of REGO on the protein expression of Bcl-2 family pro-apoptotic members by western blotting analysis. Compared with the control group, REGO treatment significantly increased the expression of Bak, Bad and Bim, but had no effect on the expression of Bax and Puma (Fig. 4A). In addition, by silencing Bcl-2 family pro-apoptotic members, we found that silencing Bim, Bak or Bax inhibited REGO-induced cytotoxicity (Fig. 4B). The consistent result was observed in HCT116 cells (Fig. S2I–K). To investigate the role of Bim, Bax and Bak in REGO-induced apoptosis, we constructed Bim-knockout (KO Bim), Bak-knockout (KO Bak) and Bax-knockout (KO Bax) cell lines by CRISPR/Cas 9 technology. Western blotting analysis confirmed the genetic deletion of Bim, Bax and Bak (Fig. S3A). CCK-8 was used to evaluate the viability of KO Bim, KO Bak and KO Bax cells treated with REGO. Compared with WT cells, KO Bim, KO Bak and KO Bax significantly inhibited REGO-induced cytotoxicity (Fig. 5C). To further determine whether Bim, Bak and Bax were involved in REGO-induced apoptosis, we determined that KO Bim, KO Bak and KO Bax inhibited REOG-induced apoptosis by flow Annexin-V/PI staining. Compared with WT cells, KO Bim, KO Bak and KO Bax inhibited significantly REGO-induced apoptosis (Fig. 4D). Furthermore, by Hoechst staining and western blotting analysis, REGO-induced upregulation of nuclear condensation and cleaved-PARP was markedly inhibited in KO Bim, KO Bak and KO Bax cells (Figs. 4E and S3F), further indicating that Bim, Bak and Bax mediate REGO-induced apoptosis. Then, we transfected YFP-Bim, stained cells with Mitotracker Deep Red 633, and used an fluorescence microscope to observed the distribution of YFP-Bim. YFP-Bim was uniformly distributed in control cells, while YFP-Bim was clearly aggregated on mitochondrial in REGO-treated cells (Fig. 4F, G). To further determines whether the activation of Bak and Bax is related to REGO-induced apoptosis, we transfected YFP-Bax in WT and KO Bim cells, stained cells with Mitotracker Deep Red 633, and observed post-transfection YFP-Bax using an fluorescence microscope. In control cells, YFP-Bax uniformly distributed, while in the REGO-treated group, YFP-Bax was significantly accumulated in mitochondria, and in KO Bim cells, the mitochondrial distribution and aggregation of YFP-Bax were significantly decreased (Fig. 4H).

Bax and Bak need to be activated by BH3-only proteins (such as Bim, Bad, tBid, and Puma) to form oligomers on mitochondria, which leads to the formation of macropores and MOMP on mitochondrial membrane, and then apoptosis [24]. Next, fluorescence resonance energy transfer (FRET) measurements were performed in live cells transfected with CFP-Bax and YFP-Bim or CFP-Bak and YFP-Bim to explore the effect of REGO on the Bim binding to Bak and Bax, respectively. Statistics were performed in at least 110 cells. Figure S4A, B shows representative cells fluorescence images of control and REGO-treated coexpressing CFP-Bax and YFP-Bim or CFP-Bak and YFP-Bim, and the corresponding pixel-to-pixel pseudo-color *E*_*D*_ and *Rc* images and the corresponding histograms (Fig. 4I). The *E*_*Dmax*_ of REGO-treated cells were 0.2197 ± 0.0206 and 0.1521 ± 0.0189, respectively, which were greater than the 0.1555 ± 0.0103 and 0.1042 ± 0.0089 of the control group, indicating that REGO promoted the binding of Bim to Bak/Bax. Despite REGO induced Bad upregulation, silencing Bad did not prevent REGO-induced loss of cell viability (Fig. 4A, B). Next, FRET measurements were performed in live cells transfected with CFP-Bcl-xl and YFP-Bad to explore the effect of REGO on the Bad binding to Bcl-xl. Statistics were performed in at least 110 cells. The *E*_*Dmax*_ of REGO-treated cells were 0.4667 ± 0.0264, respectively, which were greater than the 0.3568 ± 0.0225 of the control group. This may be explained by that Bad cann’t directly activate Bax/Bak, while REGO treatment enhances the binding of Bcl-xl to Bad (Fig. S4C, D). Taken together, these results suggest that Bim is involved in REGO-triggered Bak and Bax activation by direct interaction.

### Bax, but not Bak, recruits Drp1 to mitochondria to mediate REGO-induced mitochondrial fission

**Fig. 6:**
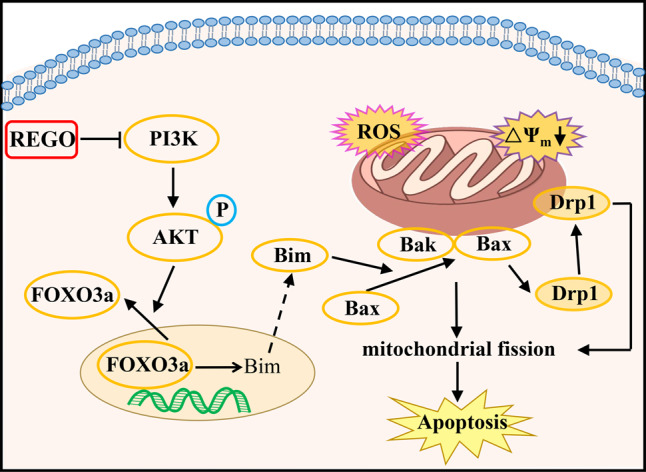
A schematic diagram of the molecular mechanism by which REGO induces apoptosis in cancer cells. REGO downregulates PI3K expression to dephosphorylate AKT that suppresses FOXO3a translocation from nucleus to cytosol, which upregulates Bim expression. Bim triggers Bax translocation to mitochondria, and mitochondrial Bax recruits Drp1 to mediate mitochondrial fission and Bax/Bak oliogomerization as well as subsequent MOMP.

A prerequisite for mitochondrial fission and apoptosis is mitochondrial translocation of Drp1 [35]. To evaluate the molecular mechanism of REGO-induced mitochondrial fragmentation, we examined the effects of REGO on the expression of the proteins related to division and fusion. Western blotting analysis showed that REGO treatment significantly increased the expression of mitochondrial division proteins Drp1 and Mff and decreased the expression of mitochondrial fusion proteins MFN1 and MFN2 (Fig. 6A). We next used the Mitotracker Deep Red 633 probe to examined the effects of REGO on mitochondrial morphology. Notably, REGO treatment cells resulted in a significant increase in the proportion of fragmented mitochondria after only 15 min compared to control cells that exhibited filamentous mitochondria (Fig. S5A). Super-resolution microscopic imaging also showed that REGO caused mitochondrial swelling (Fig. S5C), indicating that REGO impaired mitochondrial function.

We next observed the distribution of YFP-Drp1 by transfecting cells with YFP-Drp1, staining cells with Mitotracker Deep Red 633. In control group (WT), YFP-Drp1 was evenly distributed, and YFP-Drp1 was obviously translocated to mitochondria after REGO treatment (Fig. 5B). We performed the same experiment in KO Bax and KO Bak cells and found that YFP-Drp1 in KO Bak group was obviously translocated to mitochondria after REGO treatment, while YFP-Drp1 in KO Bax group was evenly distributed among cells (Fig. 5C), demonstrating that it is Bax and not Bak that raises Dpr1 translocated to mitochondria. To investigate the role of Drp1 in REGO-induced mitochondrial fission and apoptosis, we constructed gDrp1-transfected (gDrp1) cell lines by CRISPR/Cas 9 technology. Western blotting analysis confirmed the genetic deletion of Drp1 (Fig. 5D). Next, we stained cells with Mitotracker Deep Red 633 and observed mitochondrial morphology using a fluorescence microscope. Compared with the filamentous mitochondria in the WT group and the gDrp1 group, the proportion of cells with mitochondrial fragmentation in the gDrp1 group was significantly reduced after REGO treatment (Fig. 5E, F). To further explore the effect of Drp1 on REGO-induced apoptosis, we detected cell viability in gDrp1 cells by CCK-8 assay. As shown in Fig. 5G, gDrp1 significantly inhibited REGO-induced cytotoxicity. Although Bax recruits Drp1, KO Bax does not affect Drp1 protein expression (Fig. S4E). These findings indicate that mitochondrial translocation of Drp1 is involved in REGO-mediated mitochondrial fission and apoptosis.

## Discussion

We report for the first time that the PI3K/AKT/FOXO3a signaling pathway plays a key role in REGO-induced intrinsic apoptosis in breast cancer MCF-7 cells. Most importantly, we discovered that the BH3-only protein Bim acts as a key initiator to directly activate Bax and Bak, and mediate REGO-induced intrinsic apoptotic pathway. In addition, Bax, not Bak, recruited Drp1 to mediate REGO-induced mitochondrial rupture and apoptosis.

Our findings that REGO inhibits the expression of PI3K and its downstream regulator p-AKT (Fig. 3A, B) and SC79 not only restores REGO-induced proliferation and migration of cancer cells but also inhibits REGO-induced apoptosis (Fig. 3E–G) demonstrate that AKT plays an important role in REGO-induced apoptosis. When PI3K is activated by growth factors and tyrosine kinases, it causes AKT to be phosphorylated [36]. p-AKT can enhance tumor cell growth and anti-apoptosis as well as invasion by increasing the expression of cell cycle, survival and invasion-related proteins in cancer cells [37]. REGO inhibits tyrosine kinase receptors (e.g., PDGFR and VEGFR), both of which will activate PI3K signaling [38, 39]. Previous studies have shown that REGO inhibits AKT phosphorylation in hepatocellular carcinoma and sarcoma and reduced PI3K and p-AKT expression of both HCC and neuroblastoma cells [40]. REGO reduces PI3K/AKT expression in colorectal, lung, breast, kidney and brain cancer cells both in vivo and in vitro, and in combination with the PI3K inhibitor PX-866 in a greater-than-additive manner to synergistically kill tumor cells [41]. Consistent with our findings, REGO inhibited proliferation in all the cell lines we used (Fig. 1A), and SC79 restored the proliferation and migration ability of cells inhibited by REGO (Fig. 3E–G). On the other hand, the expression of PI3K, p-AKT was reduced by REGO, further indicating that REGO inhibits the PI3K/AKT signaling pathway.

The facts that REGO-induced nuclear localization of FOXO3a is significantly inhibited by SC79 and SC79 inhibits REGO-induced Bim upregulation (Fig. 3B) demonstrate that FOXO3a is an important downstream target of the REGO-induced PI3K/AKT pathway. AKT is a negative regulator of FOXO3a activity, and activation of AKT causes FOXO protein phosphorylation and promotes its entry into the nucleus [42]. In nucleus, FOXOs mediate transcription of a wide array of target genes involved, including the Fas ligand (FasL), TRAIL (TNF-related apoptosis-inducing ligand) and TRADD (TNF receptor type 1 associated death domain), as well as intracellular apoptotic components such as Bim and Bcl-6 [15, 16]. Idelalisib promotes Bim expression by inhibiting PI3K/AKT and thereby promoting FOXO3a nuclear localization in HCC cells [43]. Nuclear localization activation of FOXO3a increases cysteine aspartase activity and promotes apoptosis in endothelial and leukemic cells [44]. Withaferin-A inhibition of AKT promoted nuclear localization of FOXO3a and induced apoptosis in prostate cancer cells, and Withaferin-A-induced apoptosis in prostate cancer cells was significantly affected when AKT was overexpressed or FOXO3a was silenced [45]. Piperlongumine inhibits AKT phosphorylation and promotes FOXO3a nuclear localization and its association with the Bim gene promoter assembly, thereby activating Bim, leading to intrinsic apoptosis in Hela, MCF-7, and MGC-803 cells [46]. Crocin induced FOXO3a nuclear localization and elevated expression of Bim and PTEN (FOXO3a target genes), which mediated apoptosis in MCF-7 and MDA-MB-231 cells [47]. FOXO3a nuclear localization mediating the cytostatic and cytotoxic effects of various chemotherapeutic agents such as paclitaxel, T63, berberine and triciribine [48, 49]. Our data showed that nuclear localization of FOXO3a under REGO treatment induced activation of the pro-apoptotic protein Bim, while gFOXO3a inhibited the expression of the pro-apoptotic protein Bim (Figs. 3C, J and S2C). Pitavastatin regulates FOXO3a nuclear localization via AKT or AMPK and induces apoptosis in oral squamous cell carcinoma via FOXO3a/Puma [50]. In contrast to this view, our results show that REGO-induced FOXO3a nuclear localization does not cause Puma upregulation, but does increase Bim expression (Fig. 3J), which also supports the previous view that FOXO3a drives the apoptotic response by upregulating the expression of the pre-apoptotic gene Bim [51]. Thus, REGO exerts its apoptotic effects may via FOXO3a-mediated Bim upregulation (Fig. 3J) and Bim translocation into mitochondria (Fig. 3G).

Our findings that KO Bim inhibited REGO-induced loss of cell viability and apoptosis (Fig. 4C, D) demonstrate the key role of Bim in REGO-induced apoptosis. Bax/Bak can be directly activated by Bid, Bim, and Puma [24]. Compared to Bim and tBid, Puma does not exhibit a strong direct interaction with Bax/Bak [52, 53]. We found that SC79 could inhibit REGO-induced Bim upregulation (Fig. 3B), which supports the previous view that FOXO3a nuclear translocation promotes the expression of the apoptotic protein Bim and the activation of apoptosis. REGO induces apoptosis in colorectal cancer cells through the release of Puma [54]. Contrary to this idea, silencing Puma did not inhibit REGO-induced cell death. In initial clinical studies, REGO appeared to be primarily a cytostatic, however reduced tumor density and cavitation were observed in majority of cancer patients treated with REGO, which is associated with REGO pro-apoptotic activity [55]. In contrast, other studies have shown that REGO induces apoptosis in bladder cancer by inducing loss of mitochondrial membrane potential and induces apoptosis in gastrointestinal and liver cancer cells mainly through Bax [56]. Bim is distributed in cytoplasm and mitochondrial species and when stimulated by apoptosis leads to the transfer of Bim to mitochondria and activation of Bax or Bak, thus promoting cell death [50]. This is consistent with our results that REGO induces loss of mitochondrial membrane potential and induces Bim translocation and upregulation of Bim expression (Figs. 2F and 4A, F). Our study showed that REGO induced Bax activation and translocation to mitochondria, which was prevented by KO Bim (Fig. 4H), further demonstrating that Bim plays a key role in REGO-induced apoptosis by triggering Bax translocation into mitochondria and Bax activation.

The facts that REGO treatment for only 15 min leads to mitochondrial fragmentation (Fig. S4A) and gDrp1 or KO Bax not only inhibit REGO-induced mitochondrial fragmentation but also KO Bax inhibits REGO-induced Drp1 recruitment (Fig. 5C) demonstrate that Bax but not Bak recruits Drp1 in REGO-induced apoptosis. Mitochondria are involved in the development and progression of cancer [57]. Since mitochondria undergo dramatic fragmentation during apoptosis, it is of great interest to study the interaction between mitochondrial dynamics and apoptosis. To determine whether mitochondria are involved in REGO-induced apoptosis, we performed a series of experiments to examine mitochondrial damage. We observed that REGO treatment induced mitochondrial fragmentation within 15 min (Fig. S4A). However, contrary evidence exists on the role of Drp1 in the kinetics of apoptosis: in some reports, it is argued that Drp1 causes mitochondrial fragmentation that is necessary for apoptosis, while others suggest that Drp1 and division have no or little effect on apoptosis [58, 59]. Our data suggest that Drp1 is required for REGO-induced mitochondrial fragmentation and apoptosis (Fig. 5F, G). Although the role of mitochondrial fragmentation in apoptosis remains controversial, with some reports suggesting a role for Drp1 downstream of Bax/Bak, some argue that Drp1 is not required for Bax and Bak activation and mitochondrial foci formation [60, 61]. Isorhamnetin (IH) combination is essential for mitochondrial fission and apoptosis mediated by chloroquine (CQ)/IH combination, mainly because CQ/IH causes Bax mitochondrial translocation and recruitment of Drp1 [62]. During dinitrosodiol-induced Bax-mediated apoptosis, Drp1 translocates to the mitochondria, leading to mitochondrial fission. Prudent et al. reported that when Bax and Bak are activated and form foci at the MOM, Drp1 is recruited to the mitochondria, oligomerizes and further contracts the mitochondria [63]. There is increasing evidence that Drp1 is involved in mitochondrial fission during apoptotic cell death. STS induced apoptosis in Bax/Bak double knockout young mouse kidney cells and Dpr1 was not recruited to the mitochondrial membrane [64]. In Hela cells treated with STS, Drp1 was recruited by Bax/Bak to make mitochondrial fission [65]. In apoptosis mediated with BH3 mimics A-1331852 and A-1210477 in HCT-116 cells, activation of Bak was found to play a crucial role in positive correlation with Drp1 expression [66]. Our data show that after REGO treatment of Drp1 translocated mitochondria, KO Bax inhibits REGO-induced Drp1 translocation and mitochondrial fragmentation, and KO Bak has no effect on REGO-induced Drp1 translocation and mitochondrial fragmentation (Fig. 6C). The fission protein endophilin B1/Bif-1 containing the structural domain of BAR (Bin/amphiphysin/Rvs) is known to be required for Bax activation and membrane recruitment during apoptosis, and it is also required for Drp1 oligomerization [66]. Activated Bax forms oligomers that bind to endophilin B1/Bif-1 to form stable microdomains that recruit Drp1 [67]. Therefore, it is hypothesized that REGO-induced Bax oligomerization binds to endophilin B1/Bif-1 to form stable microstructural domains that induce Drp1 recruitment, whereas Bak does not bind to endophilin B1/Bif-1.

The molecular mechanism by which REGO induces cell apoptosis is summarized in Fig. 6. REGO downregulates PI3K expression to dephosphorylate AKT that suppresses FOXO3a translocation from nucleus to cytosol, which upregulates Bim expression. Bim triggers Bax translocation to mitochondria, and mitochondrial Bax recruits Drp1 to mediate mitochondrial fission and Bax/Bak oliogomerization as well as subsequent MOMP. Although these findings provide new insights and targets for REGO-induced apoptosis, the clinical relevance of our study remains to be further determined using other preclinical models and human patient specimens from clinical trials.

## Materials and methods

### Reagents and antibodies

Regorafenib was purchased from MedChemExpress (New Jersey, USA). Staurosporine (STS), N-acetyl-l-cysteine (NAC), ROS assay Kit, JC-1 assay Kit, and caspase-3/8/9 activity assay Kits were obtained from Beyotime (Shanghai, China). Rabbit monoclonal antibodies to PI3K (4292), Bim (2933), Bax (5023), Bak (12105), Puma (98672), Bad (9268), Bcl-xl (2764), p-AKT (Ser473) (4060), AKT (9272), FOXO3a (12829), Drp1 (8570), Mff (14739), MFN1 (14739), MFN2 (11925), and PARP (9532) antibodies were purchased from Cell Signaling Technology (Massachusetts, USA). Luciferase Mycoplasma Detection Kit, mouse monoclonal antibodies tubulin, actin and GAPDH antibodies were obtained from TransGen (Beijing, China). TurbofectTM transfection reagent and Mitotracker Deep Red 633 were obtained from Thermo Fisher Scientific (Massachusetts, USA). Cell counting Kit-8 (CCK-8) and Alexa Fluor® 488 annexin V/Dead Cell Apoptosis Kit were obtained from Dojindo (Kyushu, Japan). Hoechst 33258 probe and PI probe were purchased from Sigma-Aldrich (Missouri, USA). Enhanced chemiluminescence (ECL) was obtained from Biosharp (Beijing, China).

### Constructs

The YFP-Drp1 plasmid (#45160) was purchased from Addgene (Massachusetts, USA). The GFP-FOXO3a plasmid (#p4188) was purchased from Miaoling Biotech Company (Wuhan, China). The FOXO3a CRISPR/Cas9knockout (KO) plasmids (sc-400308) was from Santa Cruz Technologies (Dallas, TX, USA). YFP-Bim was synthesized by Gene Create Company (Hong Kong, China). YFP-Bax was kindly provided by Dr. Prehn [68]. Plasmids encoding the CFP-Bcl-xl was kindly supplied by A. P. Gilmore [25]. CFP-Bak (#31501) plasmids were purchased from Addgene Company. shBax, shPuma, shBad, shBak, shBim, shBid plasmid were constructed as previously described [69]. The epiCRISPER vector was kindly provided by Dr. Wang [70]. Oligonucleotides targeting the guide RNAs of Bim, Bax, Bak and Drp1 were cloned into epiCRISPER vector species. The plasmids targeting Bim, Bax, Bak and Drp1 were transfected into cells. After 24 h of transfection, DMEM was replaced with selection medium containing 1 µg/ml puromycin and 10% fetal bovine serum. The knockdown effects of Bim, Bax, Bak and Drp1 were confirmed by western blotting. The primer sequences used to produce epiCRISPER-KO Bim were as follows:

Forward: CCGGCCCAAGAGTTGCGGCGTAT

Reverse: AACA TACGCCGCAACTCTTGGGC

The primer sequences used to produce epiCRISPER-KO Bax were as follows:

Forward: CCGAGCGAGTGTCTCAAGCGCAT

Reverse: AACA TGCGCTTGAGACACTCGCT

The primer sequences used to produce epiCRISPER-KO Bak were as follows:

Forward: CCGGCATGAAGTCGACCACGAAG

Reverse: AACCTTCGTGGTCGACTTCATGC

The primer sequences used to produce epiCRISPER-KO Drp1 were as follows:

Forward: CCGGCTGCCTCAAATCGTCGTAG

Reverse: AACCTACGACGATTTGAGGCAGC

### Cell lines and culture

The MCF-7 cells, HCT116 cells, Hela cells, A549 cells, U87 cells, and CAL-27 cells were obtained from the tumor cell bank of the Chinese Academy of Medical Science and cultured in Dulbecco’s modified eagle medium (DMEM) containing 10% fetal bovine serum (Gibco, USA), 100 U/ml penicillin G and 100 μg/ml streptomycin in a humidified atmosphere with 5% CO_2_ at 37 °C. All cell lines were passaged early and cryopreserved and maintained in culture for <3 months, treated regularly for mycoplasma contamination using the luciferase mycoplasma detection kit.

### Cell viability assay

CCK-8 was used to measure cellular dehydrogenase activity. Dehydrogenase activity can indirectly reflect cell viability. Cells (1 × 10^4^ cells/well) were seeded in DMEM medium (100 μl/well) into 96 well plates with five replicate wells for 24 h at 37 °C, 5% CO_2_, then treated with different concentrations of REGO for 24 h. Then 100 μl DMEM containing 10 μl CCK-8 was added to each well and incubated at 37 °C, 5% CO_2_, for 1 h. The absorbance was measured at 450 nm using auto-microplate reader. Cell viability was normalized compared to the control group.

### Hoechst 33258 staining

Seed 200 μl of cells (1 × 10^4^ cells/well) into a confocal dish. The cells were washed three times with 1 × PBS and then stained with Hoechst 33258 (50 μg/ml at 37 °C, 5% CO_2_ for 20 min protected from light). 1 × PBS was washed three times and imaged by fluorescence microscopy (Carl Zeiss, Oberkochen, Germany).

### Flow cytometric analysis

According to the recommended protocol, annexin V-FITC binding was performed. Cells (1.0 × 10^6^ cells/well) were seeded in 6 well plates and treated with REGO for 24 h. The cells were digested and harvested by trypsin and washed with cold 1 × PBS. Then stained with 2 μl of propidium iodide (PI) and 2 μl of Annexin V-FITC in cold 1 × binding buffer for 15 min at room temperature in the dark. After staining, the cells were washed once with cold 1 × binding buffer, then cells were immediately transferred to 4 °C and the samples were analyzed immediately using a FACScan cytofluorometer (BD Biosciences, New Jersey, USA).

### Western blotting analysis

Cells (1.0 × 10^6^ cells/well) were seeded in 6 well plates. Washed once with cold 1 × PBS, then incubation with RIPA lysate containing protease inhibitor and phosphatase inhibitor and obtain total protein. The supernatant was collected by centrifugation at 14,000 rmp for 10 min at 4 °C. Protein assay by bicinchoninic acid (BCA). Proteins were separated by 12% sodium dodecyl sulfate-polyacrylamide gel electrophoresis (SDS-PAGE) gel and transferred to a 0.22 μm PVDF membrane. After incubation with 5% skim milk powder for 2 h at room temperature, the membranes were incubated with antibodies overnight at 4 °C. After washing three times with TBST, the membranes were incubated with HRP rabbit or mouse antibodies for 1.5 h at room temperature. The bands were visualized most frequently using ECL chemical substrate.

### Measurement of mitochondrial membrane potential

Mitochondrial membrane potential changes (MMP, ΔΨ) were detected by a 5,5’,6,6’-tetrachloro-1,1’,3,3’-tetraethylbenzimidazole carbon iodide (JC-1) fluorescent probe. Cells (1 × 10^4^cells/well) were inoculated in confocal dishes for 24 h and treated with the indicated drugs. Added 200 μl of 0.5 mM JC-1 probe and incubate for 20 min at 37 °C, 5% CO_2_ protected from light. Washed three times with 1 × PBS, add 200 μl DMEM, and detect JC-1 aggregates and JC-1 monomers by fluorescence microscopy at 585/590 and 514/530 nm Ex/Em wavelengths. The MMP was determined by the red-green fluorescence ratio.

### Quantitative FRET imaging and ED saturation assay

Briefly, quantitative FRET imaging was performed on a fluorescence microscope (Axio Observer 7, Carl Zeiss, Oberkochen, Germany) and donor-centric FRET efficiency (*E*_*D*_) and acceptor-donor ratio (*Rc*) were measured by the FRET method as described previously [70–72].

CFP-tagged protein plasmid were co-transfected with different concentrations of YFP-tagged protein plasmid in cells as described previously [71]. Cells were analyzed by selecting fluorescent signals higher than three times the backgrounds value and less than the saturation value of the fluorescent signal (65,535). The concentration ratio of total acceptor to donor was plotted against the *E*_*D*_ values. The saturation binding curves were fitted with the origin function: *E*_*D*_ = *E*_*Dmax*_ × *Rc*/(*K*_*d*_ + *Rc*). Where *E*_*Dmax*_ and *K*_*d*_ are the maximum *E*_*D*_ and the relative equilibrium dissociation constant corresponding to the saturation of the acceptor binding site, respectively [73].

### Detection of intracellular ROS

An oxidation-sensitive fluorescent probe (DCFH-DA) was used to detected intracellular ROS. Cells (1 × 10^5^ cells/well) were seeded in 6 well plates with the indicated drug treatments. Cells were washed once with 1 × PBS according to the manufacturer’s instructions and 10 µM/l DCFH-DA was incubated for 30 min at 37 °C, 5% CO_2_ protected from light. Detection by flow cytometer.

### Wound healing assay

Cells (1 × 10^5^ cells/well) were seeded in 6 well plates. When the confluence was 90%, 10 μl sterile pipette tip was used as a tool to create the linear scratch. Then washed once with serum-free DMEM to remove cellular debris. Images were taken with a fluorescence microscope (Carl Zeiss, Oberkochen, Germany) at 0 and 24 h. The ratio of the distances between the 24 and 0 h wound edges was used to analyzed the cell migratory ability.

### Statistical analyses

Data were analyzed using Graph Pad Prism 6 software (Graph Pad Software, Inc, La Jolla, CA, USA) and all data were expressed as the mean ± SD of at least three independent experiments. Student’s *t* test and two-way analysis were used for statistical analysis. Values of *p* < 0.05 were considered statistically significant.

## Supplementary information

Supplemental Table 1

Supplemental Figure legends

Supplemental Figure 1

Supplemental Figure 2

Supplemental Figure 3

Supplemental Figure 4

Supplemental Figure 5

Original Data File

Original Data File

Original Data File

## Data Availability

All data generated or analyzed during this study are included in this published article.
