# Supplementary material for: Regorafenib induces Bim-mediated intrinsic apoptosis by blocking AKT-mediated FOXO3a nuclear export
Source: Cell Death Discov. 2023 Jan 31;9:37. doi: 10.1038/s41420-023-01338-9 (PMC9889785; doi:10.1038/s41420-023-01338-9)
Supplement: Supplementary file 1 — Supplemental Table 1 [file 41420_2023_1338_MOESM1_ESM.docx]

| **Table S1 Primers for RT-PCR** | | |
| --- | --- | --- |
| **Target gene for**  **Amplification** | **Direction of**  **Primer** | **Sequence (5′→ 3′)** |
| IGFBP1 | Forward | CCCAGAGAGCACGGAGATAA |
|  | Reverse | AGAGCCTTCGAGCCATCATA |
| p27 | Forward | AGACGGGGTTAGCGGAGCAA |
|  | Reverse | TCTTGGGCGTCTGCTCCACA |
| BIM-EL | Forward | GAGCCACAAGGTAATCCTGAA |
|  | Reverse | ATACCCACTGGAGGATCGAG |
| actin | Forward | GACGGCCAGGTCATCACTAT |
|  | Reverse | ATGCCACAGGATTCCATACC |
| GAPDH | Forward | ACCACAGTCCATGCCATCAC |
|  | Reverse | TCCACCACCCTGTTGCTGTA |
| SOD2 | Forward | GTTCAATGGTGGTGGTCATATCA |
|  | Reverse | GCAACTCCCCTTTGGGTTCT |
| BCL 6 | Forward | ACACATCTCGGCTCAATTTGC |
|  | Reverse | AGTGTCCACAACATGCTCCAT |
| FOXO1 | Forward | AAGAGCGTGCCCTACTTCAA |
|  | Reverse | CTGTTGTTGTCCATGGATGC |
| FOXO4 | Forward | GGGAAAAGGCCATTGAA |
|  | Reverse | TTCAGCATCCACCAAGA |
