# Supplementary material for: Regorafenib induces Bim-mediated intrinsic apoptosis by blocking AKT-mediated FOXO3a nuclear export
Source: Cell Death Discov. 2023 Jan 31;9:37. doi: 10.1038/s41420-023-01338-9 (PMC9889785; doi:10.1038/s41420-023-01338-9)
Supplement: Supplementary file 2 — Supplemental Figure legends [file 41420_2023_1338_MOESM2_ESM.docx]

**Supplemental Figure legends**

**Supplemental Fig.1** REGO induces mROS in MCF-7 cells. (A) SOD2 expression after treatment with REGO, GAPDH was used as control. (B) Representative fluorescent images of MitoSOX (red) and CFP-acta (cyan) double staining in the presence or absence of REGO. (C) REGO induced mROS-dependent cytotoxicity measured by CCK-8 assay. Cells were pretreated with MitoTEMPO (20 μM) for 2 h, followed by co-treatment with REGO at 20 μM for 24 h.

**Supplemental Fig.2** REGO induces apoptosis by inhibiting the PI3K/AKT/FOXO3a signaling pathway. (A) SC79 prevents the REGO-induced loss of cells migration assessed by wound healing assay. (B) Flow cytometry analysis on the inhibitor effects of SC79 on REGO-induced apoptosis. (C) Bim, p27^KIP1^ and SOD2 expression after treatment with REGO, GAPDH was used as control. (D) Representative fluorescence images of WT or gFOXO3a cells expressing YFP-Bim in the presence or absence of REGO (600×). (E) FOXO3a immunofluorescence. DAPI (blue), FOXO3a (red). (F) FOXO1 and FOXO4 expression, GAPDH was used as control. (I) Western blotting analysis on REGO-regulated the expression of apoptosis-related proteins as indicated. (J and K) Representative fluorescence images of cells expressing GFP-FOXO3a in the presence or absence of REGO.

**Supplemental Fig.3** Bim is involved in REGO-triggered Bak and Bax activation by direct interaction. (A) Western blotting analysis of the effect of silencing apoptosis-relatedd proteins. (B) Flow cytometry analysis on Bax, Bak and Bim played an important role in REGO-induced apoptosis. (C) Western blotting analysis confirmed CRISPR/Cas9-mediated knockout of Bax, Bak and Bim. (D) REGO-induced nuclei condensation detected by Hoechst 33258 staining (600×). (E) Representative fluorescence images of WT or KO Bim cells expressing YFP-Bim in the presence or absence of REGO (600×). (F) Western blotting analysis on Bax, Bak and Bim were involved in REGO-triggered cleavage of PARP.

**Supplemental Fig.4** Bim is involved in REGO-triggered Bak and Bax activation by direct interaction. (A and B) Quantitative FRET measurements in living cells coexpressing CFP-Bak and YFP-Bim or CFP-Bax and YFP-Bim and the corresponding *E_D_*-*R_C_* plot. (C) REGO up-regulated Bcl-xl by Western blotting analysis. Cells were treated with REGO for 24 h. (D) Quantitative FRET measurements in living cells coexpressing CFP-Bcl-xl and YFP-Bad, and the corresponding *E_D_*-*R_C_* plot from at least 90 cells.

**Supplemental Fig.5** REGO induces mitochondrial fission. (A and B) MCF-7 cells were treated with REGO (20 μM), mitochondrial morphology was observed by MitoTracker Deep Red staining and fluorescence microscopy (600×). (C) REGO-induced mitochondrial swelling. Cells were stained with Mito-tracker Deep Red staining after treatment with 20 μM REGO for 24 h. (D) Representative fluorescence images of WT or KO Bax or KO Bak cells expressing YFP-Drp1 in the presence or absence of REGO (600×). (E) Western blotting analysis of Drp1 expression in WT or KO Bax cells. ****p< 0.0001.
