# Supplementary figures and images for: Regorafenib induces Bim-mediated intrinsic apoptosis by blocking AKT-mediated FOXO3a nuclear export
Source: Cell Death Discov. 2023 Jan 31;9:37. doi: 10.1038/s41420-023-01338-9 (PMC9889785; doi:10.1038/s41420-023-01338-9)

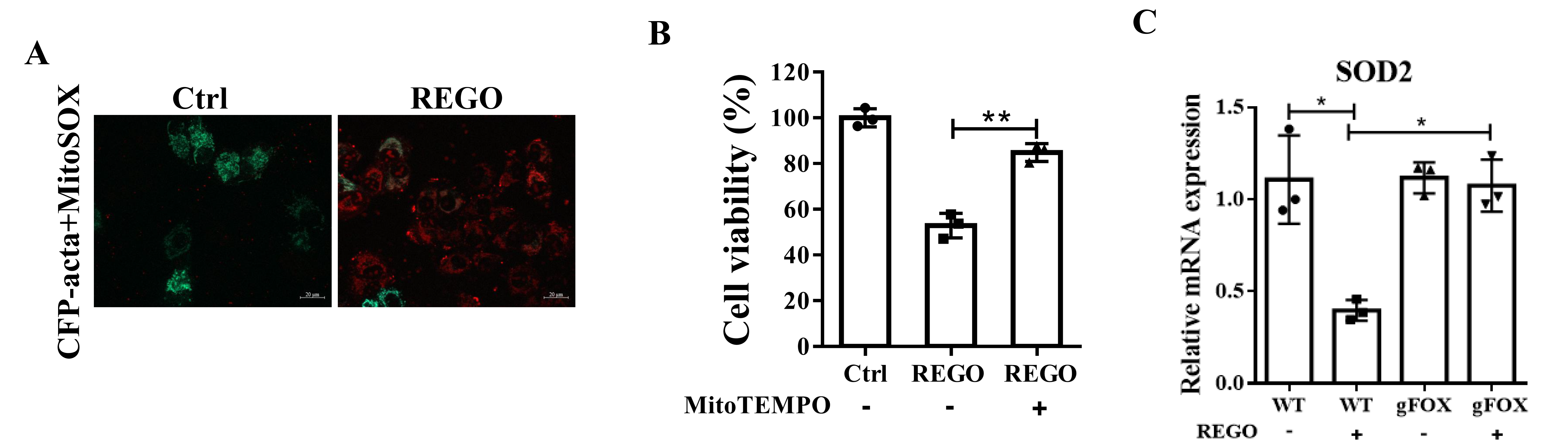

Supplement: Supplementary file 3 — Supplemental Figure 1 [file 41420_2023_1338_MOESM3_ESM.tif]

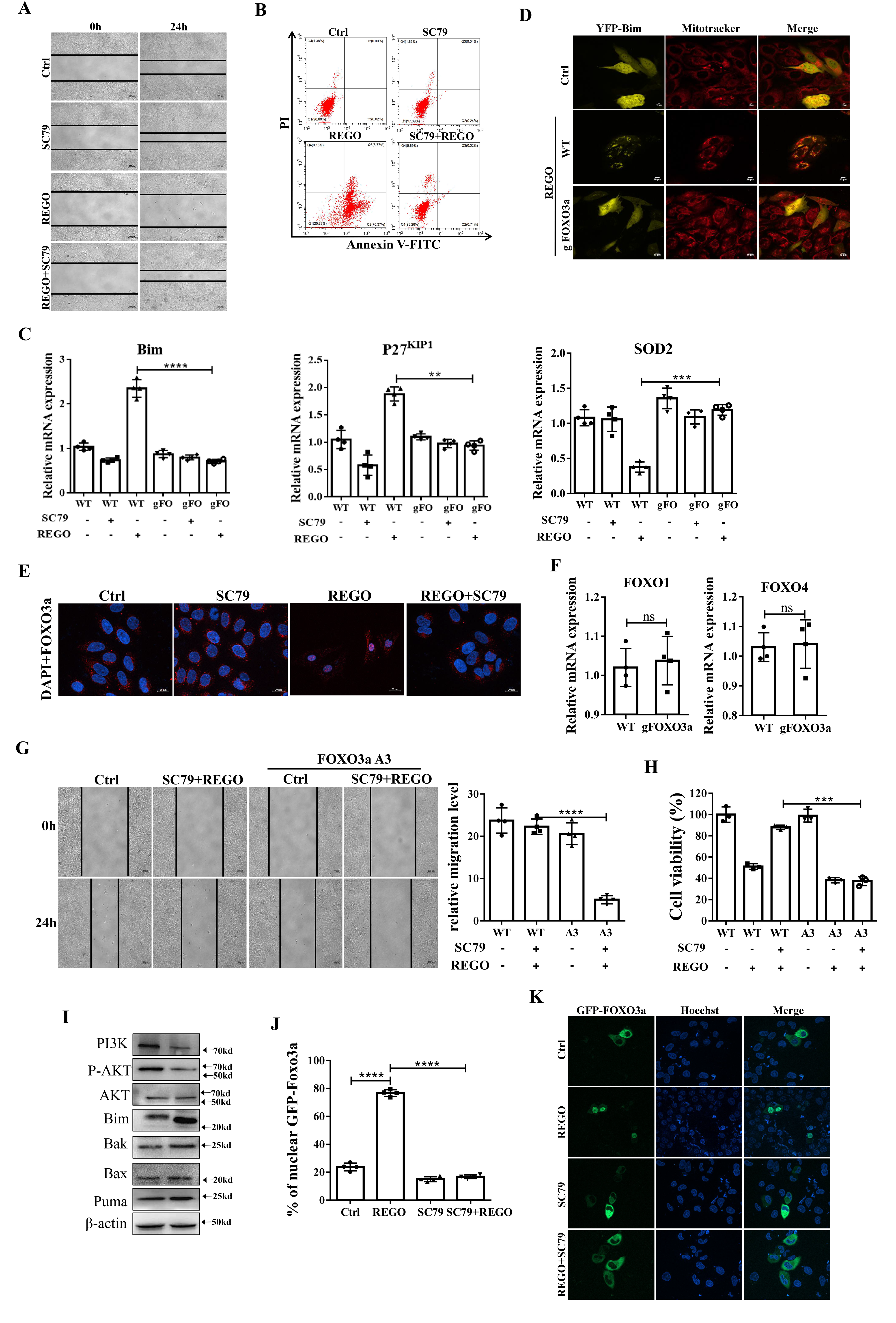

Supplement: Supplementary file 4 — Supplemental Figure 2 [file 41420_2023_1338_MOESM4_ESM.tif]

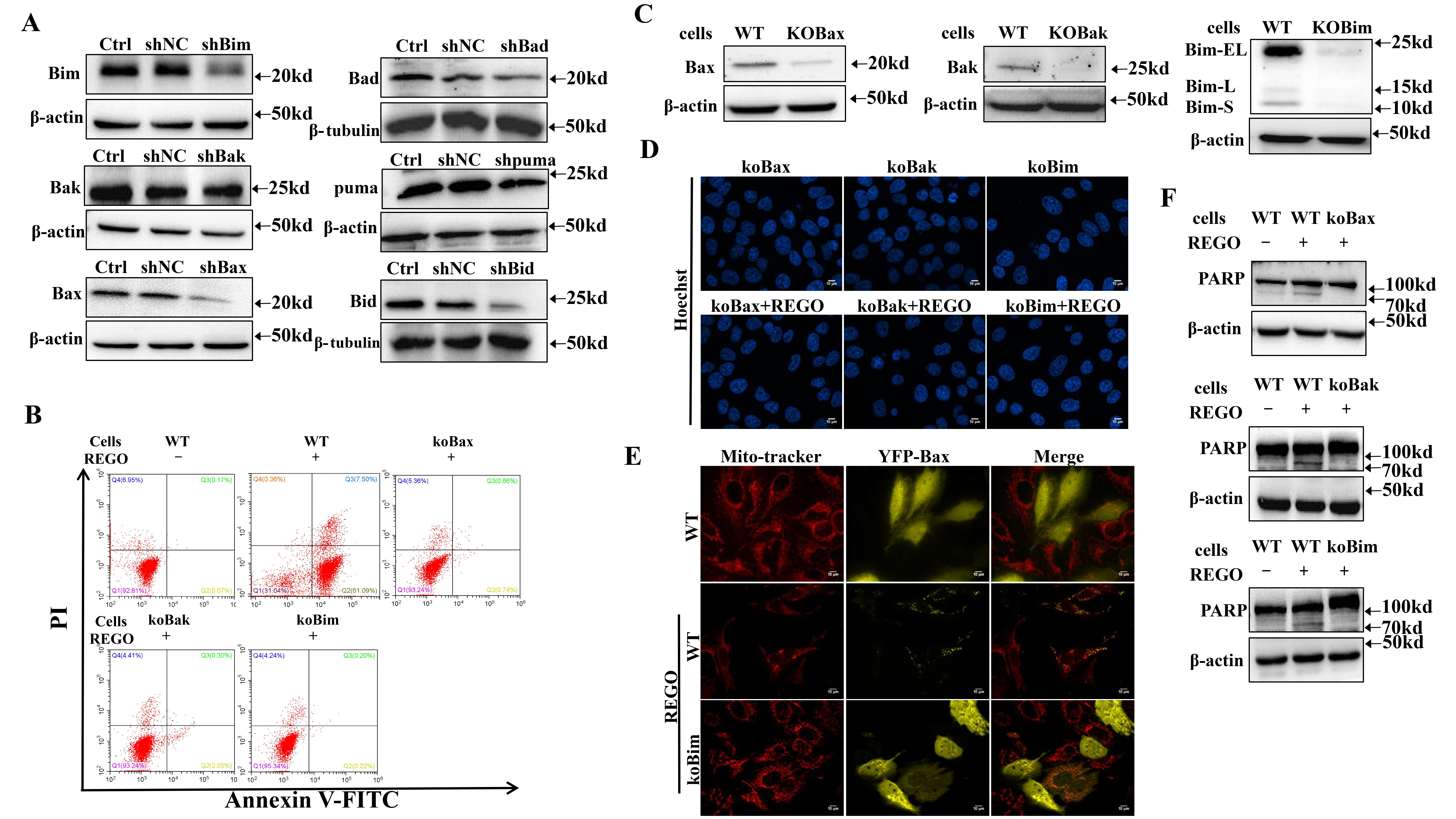

Supplement: Supplementary file 5 — Supplemental Figure 3 [file 41420_2023_1338_MOESM5_ESM.tif]

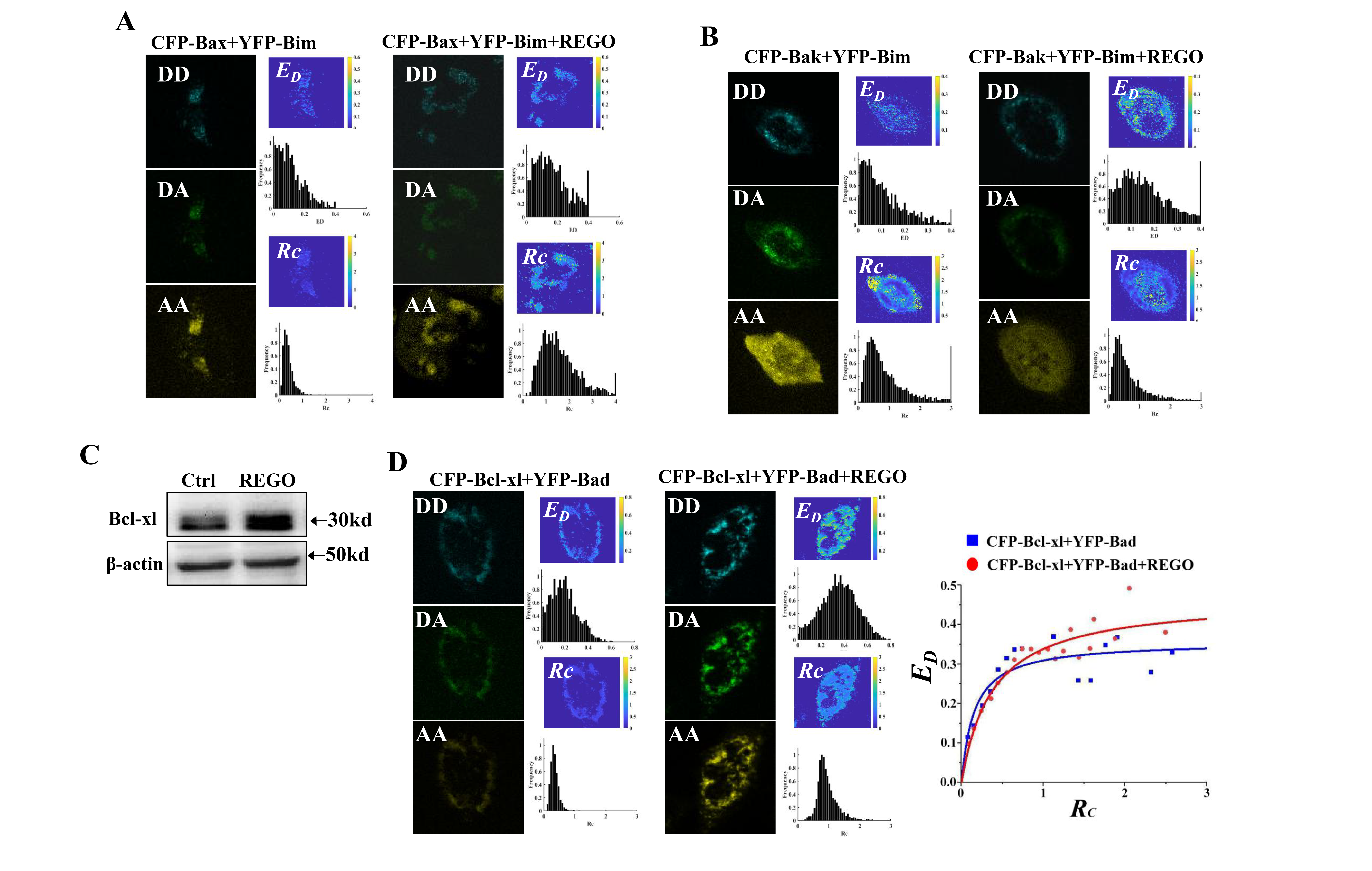

Supplement: Supplementary file 6 — Supplemental Figure 4 [file 41420_2023_1338_MOESM6_ESM.tif]

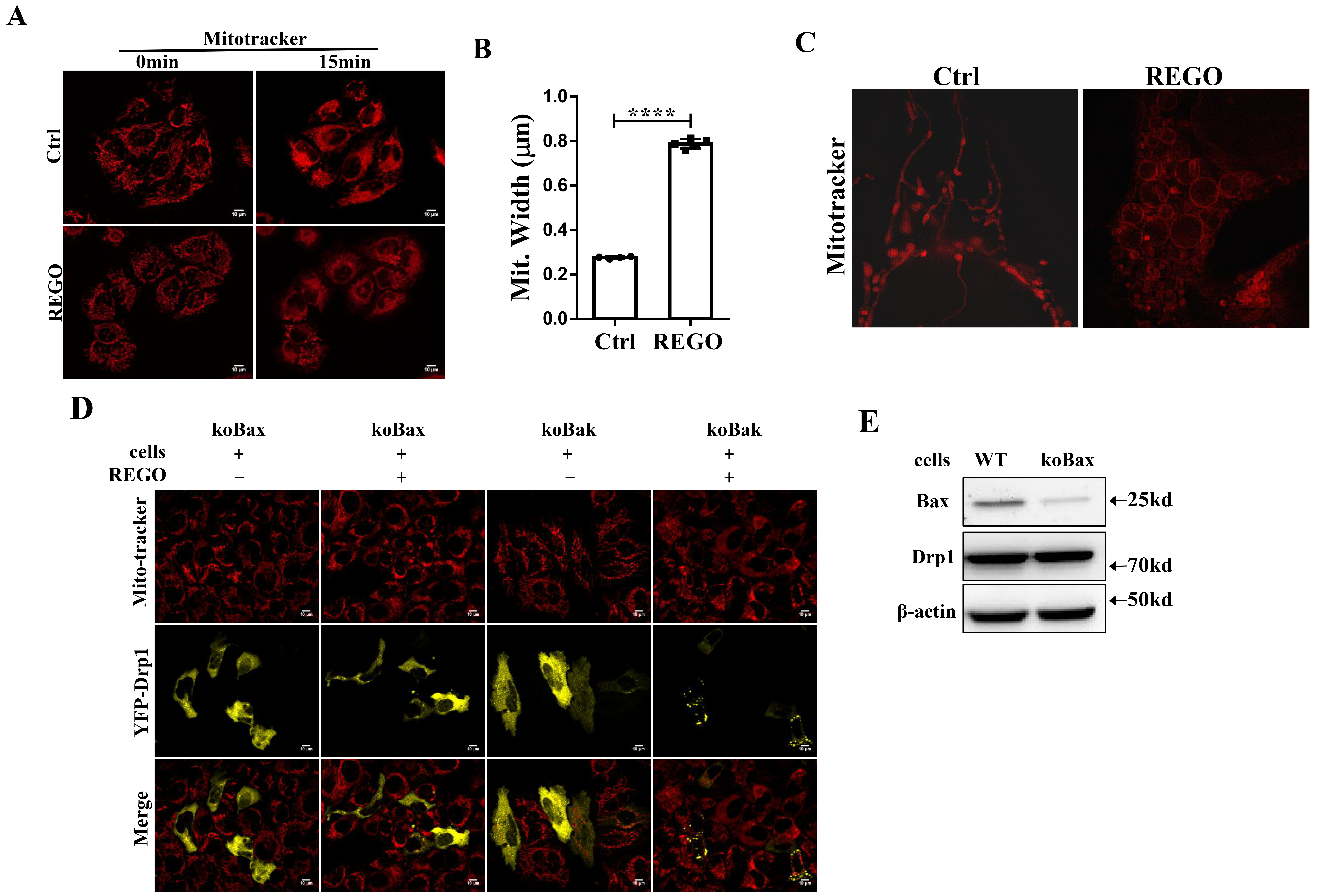

Supplement: Supplementary file 7 — Supplemental Figure 5 [file 41420_2023_1338_MOESM7_ESM.tif]

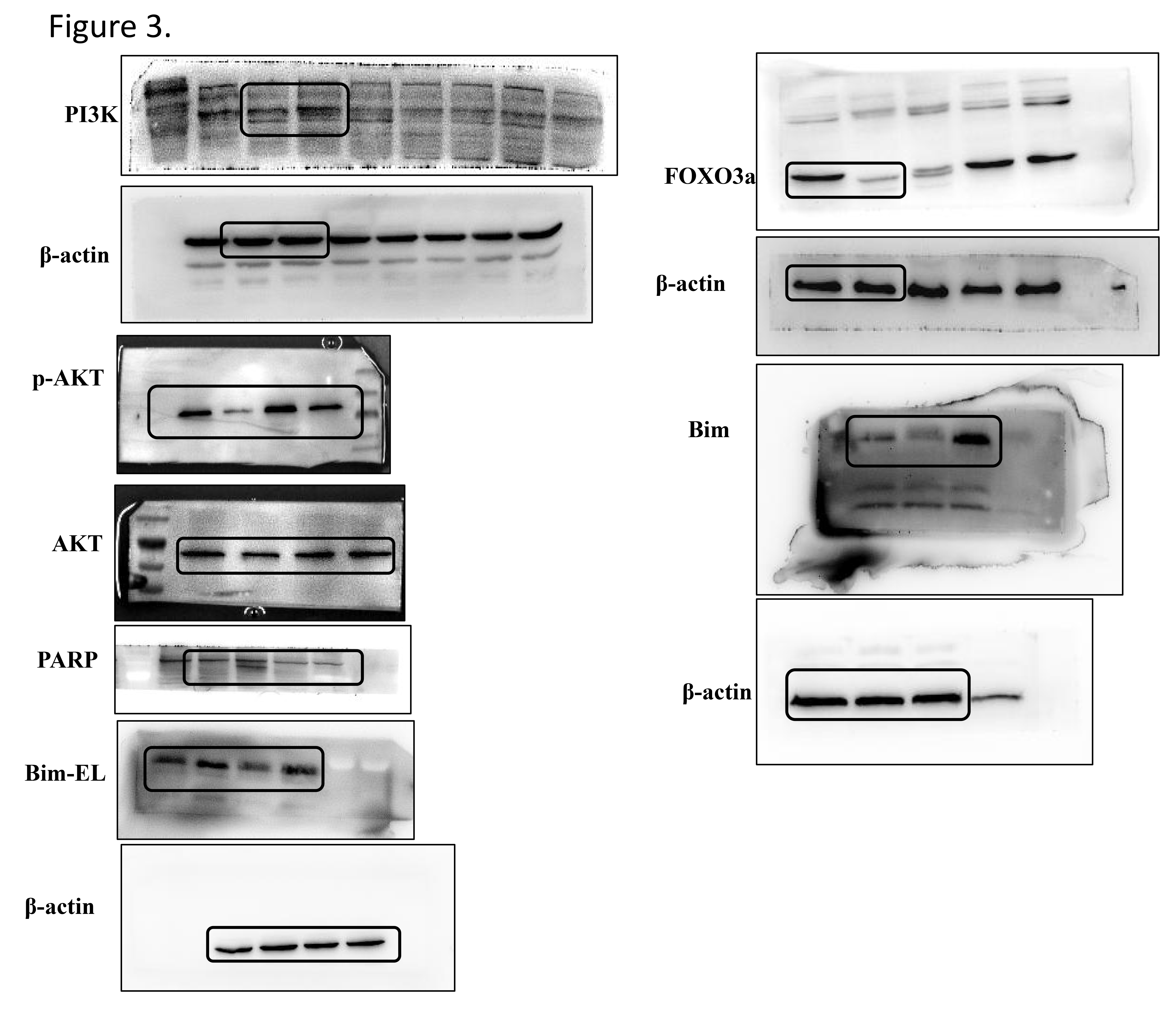

Supplement: Supplementary file 8 — Original Data File [file 41420_2023_1338_MOESM8_ESM.tif]

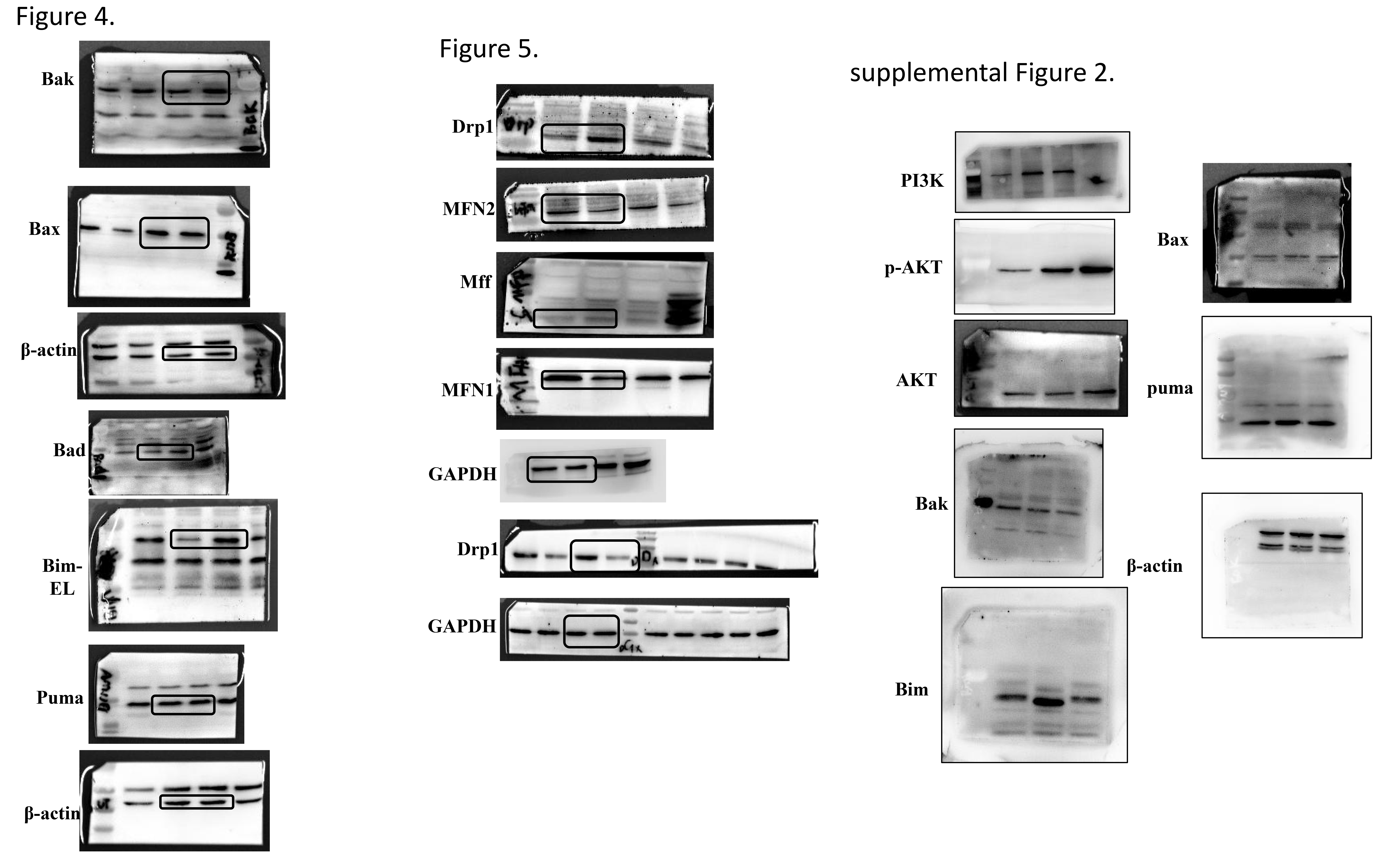

Supplement: Supplementary file 9 — Original Data File [file 41420_2023_1338_MOESM9_ESM.tif]

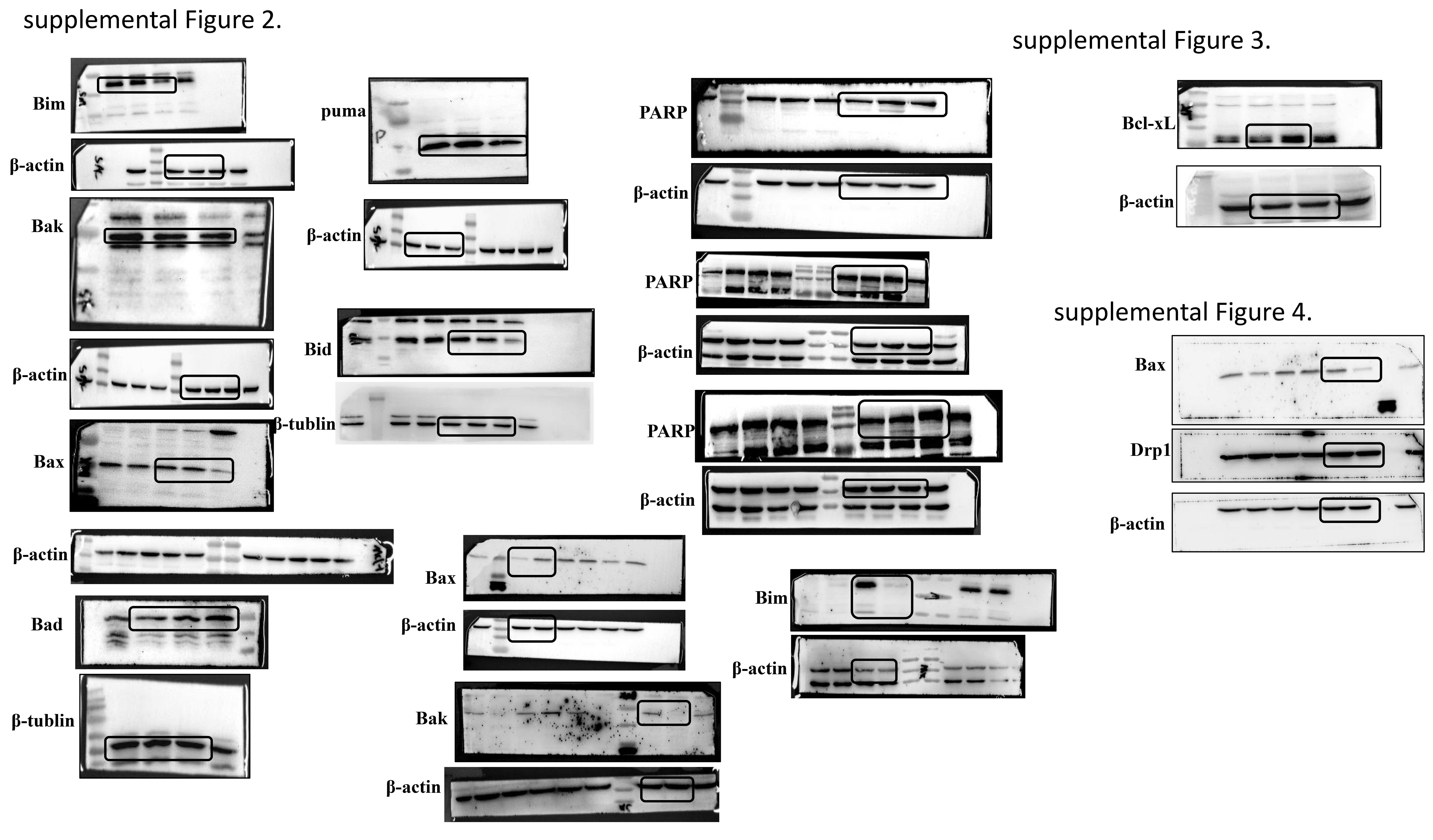

Supplement: Supplementary file 10 — Original Data File [file 41420_2023_1338_MOESM10_ESM.tif]
